# Chinese Version of the mHealth App Usability Questionnaire for Health Care Providers: Cross-Sectional Translation and Validation Study

**DOI:** 10.2196/86783

**Published:** 2026-07-29

**Authors:** Mengxia Chen, Wenjun Gao, Yijie Pan, Mengdi Wang, Bing Yu, Dongmei Li, Huanhuan Hu, Jing Zheng, Lingjuan Zhang, Yuan Gao

**Affiliations:** 1 Department of Nursing, the First Medical Center of Chinese People’s Liberation Army General Hospital Beijing China; 2 Nursing College, Naval Medical University Shanghai China; 3 Education and Scientific Research Department of Clinical Nursing, Changhai Hospital Shanghai China; 4 Department of Emergency and Critical Care Medicine, The 961th Hospital of Joint Logistics Support Force Qiqihaer China; 5 Neurovascular Center, Changhai Hospital Shanghai China

**Keywords:** mobile health app, mHealth app, questionnaire translation, usability, health care provider, Chinese

## Abstract

**Background:**

With the rapid proliferation of mobile health (mHealth) apps in clinical practice, ensuring their usability for health care providers is critical to optimize workflow integration and service delivery. The mHealth App Usability Questionnaire (MAUQ) is a validated tool for assessing usability, but its applicability in Chinese health care settings remains untested. Cross-cultural adaptation and validation of the MAUQ for interactive apps are essential to address this gap.

**Objective:**

This study aimed to translate the MAUQ (for interactive apps for health care providers) into Chinese (MAUQ-C), adapt it to the Chinese health care context, and validate its psychometric properties for future mHealth research and use in China.

**Methods:**

This cross-sectional study followed international guidelines for cross-cultural adaptation, including forward translation, backward translation, expert review, and pretesting. A convenience sample of 382 health care providers (eg, physicians and nurses) using mHealth apps was recruited from 2 hospitals in China. Reliability was evaluated using the Cronbach α, test-retest reliability, and split-half reliability. Validity was assessed through content validity index, exploratory factor analysis, and confirmatory factor analysis.

**Results:**

The MAUQ-C retained the original 21-item structure and 3 dimensions. Content validity index values ranged from 0.833 to 1.000 (Scale-Content Validity Index/Average=0.992). Exploratory factor analysis identified a 3-factor model explaining 63.48% of the total variance. Confirmatory factor analysis confirmed a good model fit (minimum discrepancy/df=1.810; cumulative fit index=0.950; root-mean-square error of approximation=0.058). The Cronbach α for the total scale was 0.919, with dimension-specific values of 0.928, 0.877, and 0.886. Test-retest reliability was 0.881, and split-half reliability was 0.726. These results confirm that the MAUQ-C effectively measures usability across the dimensions of usability and satisfaction, system information arrangement, and efficiency.

**Conclusions:**

The MAUQ-C is a reliable and valid instrument for evaluating mHealth app usability among Chinese health care providers. It can serve as a critical tool to guide the development and optimization of mHealth solutions tailored to the Chinese health care system.

## Introduction

The substantial user base for internet medical services of 418 million by 2024 in China provides a critical foundation for mobile health (mHealth) [1]. The World Health Organization defines mHealth as the “medical and public health practice supported by mobile devices, such as mobile phones, patient monitoring devices, personal digital assistants, and other wireless devices” [2]. Driven by advancing public health literacy and internet technology, this sector has experienced explosive growth and become extensively integrated into personal health management ecosystems [3,4], where mHealth apps typically offer multifaceted functionalities, including monitoring and recording health data, disease management [5], and medical guidance [6], among others [7].

However, nearly half a million mHealth apps developed remain underused [8], largely due to the absence of effective usability evaluations. This gap contributes to skepticism among clinicians, scholars, and patients regarding the reliability of mHealth programs [9]. High-usability mHealth apps typically exhibit 5 core attributes: efficiency, favorable user perception, ease of learning, memorability, and low error rates [10]. While usability questionnaires remain the predominant method for evaluating usability, the most commonly used instruments—such as the System Usability Scale (SUS) and Post-Study System Usability Questionnaire (PSSUQ) [11]—are primarily designed for expert-based objective evaluation, not specifically to assess mHealth app usability. In addition, mHealth apps offer a unique and readily accessible platform not only for the patient but also for the health care provider. To address the crucial need for evaluating the utility and usability from both patient and health care provider perspectives, the mHealth App Usability Questionnaire (MAUQ) was crafted by Zhou et al [12] in 4 different versions categorized based on the nature of the app (interactive or stand-alone) and the intended user (patient or health care provider). The MAUQ’s unique design—differentiating between patient and health care provider versions and focusing on interactive app functionalities—makes it particularly suitable for evaluating how mHealth tools integrate into the complex clinical workflows of health care professionals. By focusing on dimensions such as system information arrangement and efficiency, the MAUQ provides actionable insights into clinical usability that broader quality assessment tools may not fully capture.

The necessity of a health care provider–specific version of the MAUQ is grounded in the fundamental differences between the use contexts of patients and health care professionals. While patients primarily focus on ease of use and personal health management, health care providers operate within complex clinical environments characterized by high cognitive load, time pressure, and the critical responsibility for clinical decision-making [3]. The patient version of the MAUQ, which emphasizes personal health outcomes and self-care, fails to capture these professional-specific dimensions such as the integration of mHealth tools into existing clinical workflows and the impact of app usability on patient safety [8]. Therefore, a validated health care provider–specific version is essential to evaluate whether an app facilitates clinical practice. Relying on the patient version of the MAUQ for professional users would lead to a misalignment between the tool’s assessment criteria and the actual professional requirements of health care providers.

The different versions of the MAUQ have been translated into different languages and validated, such as into Chinese (interactive apps, patient version) [8], Spanish (stand-alone apps, patient version) [13], Persian (interactive apps, patient version) [14], Malay (stand-alone apps, patient version) [15], and German (stand-alone apps, patient version) [16]. However, China has not yet introduced a Chinese version of the MAUQ (MAUQ-C; interactive apps for health care providers). Therefore, the present study aimed to translate and verify the health care provider version of the MAUQ for interactive apps into Chinese.

## Methods

### Study Design

This was a cross-sectional study using the MAUQ. The original English version was developed to assess the usability of mHealth apps among patients and health care providers with a strong internal consistency [12]. The MAUQ for interactive apps for health care providers was used in this study. The MAUQ contains a total of 21 items and 3 dimensions: usability and satisfaction (8 items), system information arrangement (6 items), and efficiency (7 items) scored on a 7-point Likert scale ranging from 1 (“extremely strongly agree”) to 7 (“extremely strongly disagree”). The usability of the app is the average score of all items (total score divided by the number of items). The closer the average score is to 1, the higher the app’s usability is. A sample size of 382 participants was determined to ensure a robust participant-to-item ratio, which exceeds the minimum requirement of 10:1 for exploratory factor analysis (EFA), thereby ensuring the stability of the factor structure.

### Inclusion and Exclusion Criteria

The inclusion criteria were (1) health care providers (physicians or nurses) currently working in the participating hospitals, (2) individuals who had used an mHealth app for at least 1 month to provide medical services, and (3) those who voluntarily consented to participate. Exclusion criteria were (1) individuals with less than 1 year of clinical experience and (2) those who were unable to complete the online questionnaire for technical or personal reasons.

### Translation and Adaptation Process

#### Translation

Permission from the author of the original questionnaire was obtained through email. The translation model by Brislin [17] was adopted. The first step is forward translation, where 2 translators with native English-language proficiency independently translated the original MAUQ into Chinese versions (C1 and C2). The research team then reconciled these versions through discussion with both translators to produce a consensus Chinese draft (C3). The second step is back translation, where 2 new translators independently translated C3 back into English (E1 and E2). The research team harmonized E1 and E2 into a single English version (E3). The third step is cross-validation, where the research team and all translators compared E3 with the original MAUQ to evaluate semantic equivalence. Discrepancies were resolved by refining C3. The revised MAUQ-C was finalized after incorporating all necessary amendments.

#### Experts’ Comments

The expert panel consisted of 6 professionals (4 clinical health care providers, 1 software engineer, and 1 English teacher), all of whom held a master’s degree or above and possessed intermediate or senior professional titles. Their expertise spanned clinical nursing, software engineering, and medical education, ensuring a comprehensive evaluation of the questionnaire’s content validity. On the basis of their theoretical knowledge and practical experience, each expert evaluated the accuracy of translation, content comprehension, language expression habits, and consistency with the cultural background of each item one by one. For suggestions that were not easy to understand, the researcher had an in-depth discussion with the experts and made detailed records. Finally, the group summarized all the opinions from experts. The expert panel was selected to ensure a balance between clinical practice and technical implementation. Although the panel lacked dedicated psychometricians, the experts’ deep familiarity with mHealth research and clinical nursing ensured that the items were culturally adapted and clinically relevant.

#### Prediction Test

A preliminary survey was conducted among 10 health care providers at a tertiary hospital using convenience sampling. The survey aimed to assess respondents’ understanding of the questionnaire and their perceptions of its items. Feedback and suggestions from participants were documented, and the final MAUQ-C for interactive apps was developed by integrating experts’ recommendations and respondents’ suggestions.

### Research Objects and Tools

This cross-sectional descriptive study was conducted in 1 third-class hospital. The convenience sampling method was adopted to select health care providers from a hospital who used an mHealth app (UHealth) to provide medical services. All participants gave informed consent and voluntarily took part in the study. UHealth is an app designed for the population with chronic diseases, providing comprehensive management such as medication reminders, online consultation, and disease knowledge [18]. The inclusion criteria were physicians or nurses who had ever used the UHealth app. The research tools included the MAUQ-C and health care workers’ general information, including their age, sex, marital status, educational level, and professional title.

### Verification of the Questionnaire

#### Item Analysis

The central tendency of the responses to each item was first examined. If a single response option accounted for over 80% of the total responses for an item, the item was considered to have weak discriminative power and was thus eliminated. Next, independent-sample 2-tailed *t* tests were conducted to compare mean scores between high- and low-score groups (top 27% vs bottom 27% of the total scores), with items showing no significant between-group differences (*P*>.05) being removed. Finally, Pearson correlation analysis was performed between individual item scores and the total questionnaire score, and items with poor correlation (*r*<0.30; *P*>.05) were excluded to ensure construct validity.

#### Reliability and Validity Testing

Six experts in mHealth were invited to evaluate the relevance of the questionnaire contents. A 4-point Likert scale was used to evaluate the correlation between each item (1=“not related”; 4=“strongly related”). The content validity index (CVI) was calculated. Principal component analysis and maximum variance orthogonal rotation were used for EFA. Items with a load factor of less than 0.40 were deleted [19]. The correlation matrix between each dimension and the total score of the questionnaire was tested, and the internal correlation analysis was carried out. Confirmatory factor analysis (CFA) was undertaken to verify whether the structure of the questionnaire was consistent with the theoretical structure of the original questionnaire. The reliability of the questionnaire was evaluated using the Cronbach α coefficient, test-retest reliability, and split-half reliability.

### Ethical Considerations

This study was conducted in accordance with the Declaration of Helsinki and was approved by the ethics committee of Changhai Hospital (approval CHE2023-061). All participants provided informed consent before taking part in the study. On the first page of the online survey, we provided a detailed introduction to the study and an informed consent form. Participants were required to click a box stating, “I agree to participate in this research of my own volition” to proceed. The Wenjuanxing platform automatically logged their consent, and participants were free to opt out at any time by closing the survey. To ensure privacy and confidentiality, all collected data were deidentified and stored in a secure, password-protected database accessible only to the research team. No personally identifiable information was collected or disclosed, and no compensation was provided.

## Results

### General Information

A total of 382 questionnaires were forwarded to health care workers in the hospital who used an mHealth app to provide medical care services for patients. Of these 382 health care workers, 248 (64.9%) were male, and 134 (35.1%) were female. Most users (n=363, 95%) were under 50 years of age. Table 1 provides further details.

**Table 1:** General characteristics of the study participants (N=382).

Category and classification			Participants, n (%)
**Sex**			
	Male	248 (64.9)	
	Female	134 (35.1)	
**Age (y)**			
	<30	92 (24.1)	
	30-40	184 (48.2)	
	40-50	87 (22.8)	
	≥50	19 (5)	
**Length of service (y)**			
	≤5	114 (29.8)	
	5-10	145 (38)	
	10-15	68 (17.8)	
	≥15	55 (14.4)	
**Professional rank and title**			
	Junior	53 (13.9)	
	Intermediate	208 (54.5)	
	Senior	121 (31.7)	

### Item Analysis

The results (Table 2) indicated a significant difference between the high- and low-score groups (t_211_=−14.497; *P*<.001). Items with no statistically significant difference (critical ratio<3 or *P*<.05) between the high- and low-score groups would be deleted [20]. However, the results showed that there were significant differences (critical ratio=8.594-16.613; *P*<.001) in each item between the high- and low-score groups; thus, no item was deleted.

The Pearson correlation coefficient was used to assess the correlation among 21 items. Items with a correlation coefficient below 0.4 or a *P* value below .05 would be deleted. As shown in Table 3, all the items were significant, and a positive correlation was found among the values of the assessment phase. The correlation coefficient between the score of each item and the total score of the questionnaire was calculated (0.505-0.743; *P*<.001); thus, all items were retained.

**Table 2:** Results of the item analysis.

Item	Expert evaluation (score of 1-4), mean (SD)			*t* test (*df*)		*P* value
	Low-score group (n=107)	High-score group (n=106)				
1	1.41 (0.49)	3.44 (1.42)	−14.497 (211)		<.001	
2	1.53 (0.64)	3.54 (1.38)	−14.120 (211)		<.001	
3	1.48 (0.54)	3.59 (1.36)	−15.382 (211)		<.001	
4	1.39 (0.66)	3.53 (1.43)	−14.486 (211)		<.001	
5	1.63 (0.62)	3.26 (1.49)	−10.803 (211)		<.001	
6	1.25 (0.46)	3.59 (1.44)	−16.613 (211)		<.001	
7	1.55 (0.62)	3.45 (1.35)	−13.593 (211)		<.001	
8	1.45 (0.55)	3.30 (1.28)	−14.198 (211)		<.001	
9	1.50 (0.64)	3.45 (1.46)	−12.997 (211)		<.001	
10	1.65 (0.65)	3.13 (1.28)	−11.029 (211)		<.001	
11	1.62 (0.58)	3.38 (1.22)	−13.905 (211)		<.001	
12	1.65 (0.71)	3.18 (1.37)	−10.609 (211)		<.001	
13	1.55 (0.59)	3.32 (1.32)	−13.086 (211)		<.001	
14	1.51 (0.62)	3.13 (1.41)	−11.224 (211)		<.001	
15	1.69 (0.85)	3.06 (1.27)	−9.561 (211)		<.001	
16	1.80 (0.75)	3.04 (1.27)	−8.963 (211)		<.001	
17	1.72 (0.69)	2.89 (1.26)	−8.618 (211)		<.001	
18	1.60 (0.64)	2.96 (1.14)	−11.039 (211)		<.001	
19	1.54 (0.64)	2.79 (1.31)	−9.162 (211)		<.001	
20	1.66 (0.69)	2.94 (1.21)	−9.784 (211)		<.001	
21	1.70 (0.81)	2.88 (1.20)	−8.594 (211)		<.001	

**Table 3:** Pearson correlation analysis results.

Item	Correlation coefficient	*P* value
1	0.727	<.001
2	0.656	<.001
3	0.743	<.001
4	0.685	<.001
5	0.589	<.001
6	0.696	<.001
7	0.641	<.001
8	0.697	<.001
9	0.647	<.001
10	0.543	<.001
11	0.622	<.001
12	0.505	<.001
13	0.656	<.001
14	0.510	<.001
15	0.582	<.001
16	0.594	<.001
17	0.527	<.001
18	0.606	<.001
19	0.602	<.001
20	0.594	<.001
21	0.538	<.001

### Validity Testing of the Questionnaire

As shown in Table 4, the score range of the CVI in the questionnaire was 0.833 to 1 (minimum value≥0.78) [20], indicating that the items were comprehensible, and the CVI of the total score of the questionnaire was 0.992.

**Table 4:** Content validity evaluation results of the Chinese health care provider version of the mHealth App Usability Questionnaire.

Item	Score by experts							Experts who provided a score of 3 or more, n		I-CVI^a^
	Expert 1	Expert 2	Expert 3	Expert 4	Expert 5	Expert 6				
1	4	4	4	4	4	4	6		1.000	
2	4	3	4	4	4	4	6		1.000	
3	2	3	3	4	4	4	5		0.833	
4	4	4	4	4	3	4	6		1.000	
5	4	4	3	3	3	4	6		1.000	
6	4	4	3	4	4	4	6		1.000	
7	4	4	4	4	4	4	6		1.000	
8	4	4	4	4	4	4	6		1.000	
9	4	4	4	3	4	3	6		1.000	
10	4	4	4	4	3	3	6		1.000	
11	4	4	4	3	4	3	6		1.000	
12	4	3	4	4	4	3	6		1.000	
13	4	4	4	4	4	4	6		1.000	
14	4	4	3	4	4	4	6		1.000	
15	4	4	4	3	3	4	6		1.000	
16	4	4	4	4	4	4	6		1.000	
17	4	4	4	4	4	4	6		1.000	
18	4	4	4	4	4	4	6		1.000	
19	4	4	4	4	4	4	6		1.000	
20	3	4	4	4	4	4	6		1.000	
21	4	4	4	3	4	4	6		1.000	

^a^I-CVI: item-level content validity index.

### Structural Validity

#### Exploratory Factor Analysis

The validity of the questionnaire was assessed through EFA of the values of the Kaiser-Meyer-Olkin (KMO) test, the Bartlett test of sphericity, common degree (common factor variance), load factor, and other indicators. The KMO test was conducted before factor analysis to evaluate whether the data were suitable for EFA. The KMO value of this study was 0.932 (>0.900), which indicated that the questionnaire had an acceptable validity. At the same time, the Bartlett test of sphericity was carried out to determine whether the correlation coefficient between items was significant. The value of the Bartlett test was *P*<.001, indicating that the questionnaire data were very suitable for EFA. According to the maximum variance method, the factors with eigenvalues greater than 1 were extracted from the 21 items, resulting in the extraction of 3 common factors with eigenvalues greater than 1. The cumulative contribution rate of these 3 common factors was 63.48% (>60%), indicating that the scale structure was more consistent with the theoretical structure. Detailed results are shown in Table 5.

**Figure 1:**
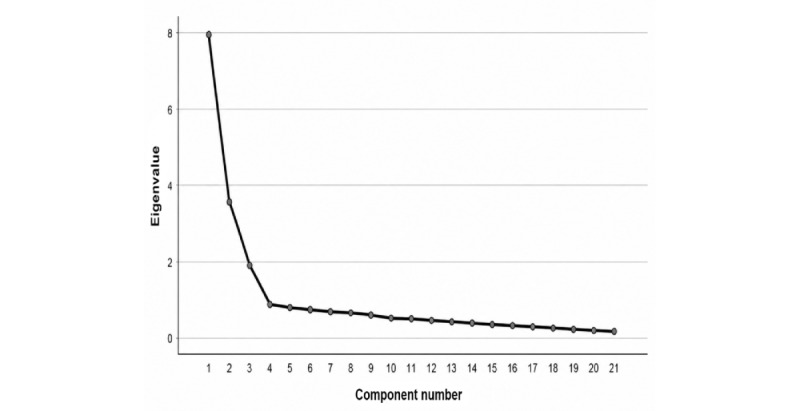
Fragmentation graph of the scale factors of the Chinese health care provider version of the mHealth App Usability Questionnaire.

The orthogonal rotation method was used to analyze the crushed stone diagram (Figure 1). The dimensions of each item were the same as those in the original version and, ultimately, resulted in an MAUQ-C for health care providers with 21 items including 3 dimensions: usability and satisfaction (8 items), system information arrangement (6 items), and usefulness (7 items).

The results of EFA are shown in Table 6.

**Table 5:** Results for the total variance interpretation.

Item	Initial eigenvalues				Squares of the extracted loadings				Squares of the transverse loadings		
	Total	Variance (%)	Cumulation (%)	Total		Variance (%)	Cumulation (%)	Total		Variance (%)	Cumulation (%)
1	7.907	37.653	37.653	7.907		37.653	37.653	5.496		26.170	26.170
2	3.554	16.924	54.577	3.554		16.924	54.577	4.129		19.660	45.830
3	1.870	8.903	63.48	1.870		8.903	63.48	3.707		17.650	63.48
4	0.797	3.796	67.276	—^a^		—	—	—		—	—
5	0.721	3.432	70.708	—		—	—	—		—	—
6	0.674	3.208	73.916	—		—	—	—		—	—
7	0.618	2.945	76.861	—		—	—	—		—	—
8	0.599	2.854	79.715	—		—	—	—		—	—
9	0.556	2.648	82.363	—		—	—	—		—	—
10	0.464	2.211	84.574	—		—	—	—		—	—
11	0.453	2.157	86.731	—		—	—	—		—	—
12	0.423	2.016	88.747	—		—	—	—		—	—
13	0.399	1.898	90.644	—		—	—	—		—	—
14	0.346	1.647	92.291	—		—	—	—		—	—
15	0.306	1.456	93.747	—		—	—	—		—	—
16	0.281	1.338	95.086	—		—	—	—		—	—
17	0.262	1.247	96.332	—		—	—	—		—	—
18	0.236	1.125	97.457	—		—	—	—		—	—
19	0.211	1.003	98.461	—		—	—	—		—	—
20	0.179	0.851	99.312	—		—	—	—		—	—
21	0.145	0.688	100.00	—		—	—	—		—	—

^a^Not applicable.

**Table 6:** Loading matrix of the scale factors of the Chinese health care provider version of the mHealth App Usability Questionnaire.

Item	Factor loadings		
	Factor 1	Factor 2	Factor 3
1	*0.862* ^a^	0.080	0.194
2	*0.842*	0.128	0.111
3	*0.828*	0.128	0.239
4	*0.776*	0.082	0.171
5	*0.787*	0.102	0.002
6	*0.853*	0.108	0.119
7	*0.827*	0.083	0.096
8	*0.673*	0.039	0.429
9	0.195	0.249	*0.749*
10	0.162	0.125	*0.649*
11	0.228	0.193	*0.689*
12	0.047	0.129	*0.771*
13	0.219	0.234	*0.767*
14	0.086	0.313	*0.676*
15	0.095	*0.706*	0.253
16	0.173	*0.661*	0.211
17	0.000	*0.743*	0.159
18	0.063	*0.687*	0.244
19	0.097	*0.848*	0.238
20	0.210	*0.711*	0.128
21	0.034	*0.764*	0.030

^a^The italicized items have a sufficiently strong and meaningful association with their assigned latent factors in the measurement model.

#### Confirmatory Factor Analysis

**Figure 2:**
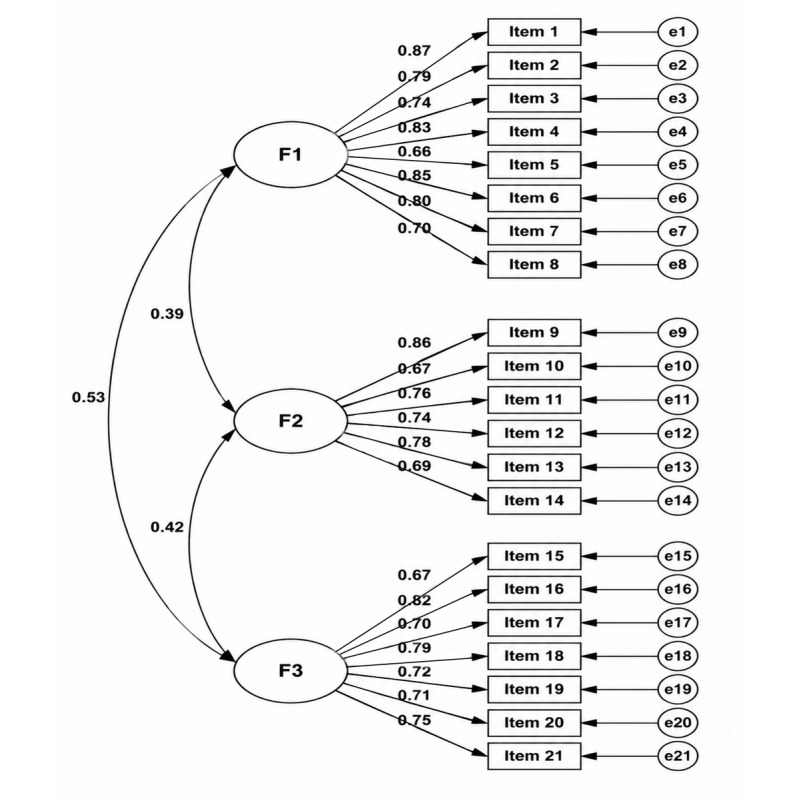
The confirmatory factor analysis model for the Chinese version of the mHealth App Usability Questionnaire. e: error, residual; F: factor.

As shown in Figure 2, in this model, the minimum discrepancy/df value was 1.810; the root-mean-square error of approximation was 0.058; the incremental fit index and cumulative fit index values were both 0.950; the goodness-of-fit index, normed fit index, and relative fit index values were 0.887, 0.895, and 0.881, respectively; and the parsimony normed fit index value was 0.793. All the goodness-of-fit indexes met the general standard, which proves that the fitness of the model was good.

#### Reliability Testing of the Questionnaire

The Cronbach α coefficient of the MAUQ-C was 0.919. A higher α value suggests greater internal reliability, and more than 0.700 is acceptable as good internal reliability. The values for test-retest reliability and split-half reliability were 0.881 and 0.726, respectively. The reliability of each dimension of the questionnaire is shown in Table 7.

**Table 7:** Questionnaire reliability analysis results.

	Cronbach α	Test-retest reliability	Split-half reliability
Dimension 1	0.928	0.759	0.933
Dimension 2	0.877	0.874	0.875
Dimension 3	0.886	0.745	0.903
Overall questionnaire	0.919	0.881	0.726

## Discussion

### Principal Findings

mHealth apps that provide health information and comprehensive management may be helpful both for patients and health care workers, and their use should be encouraged. The evaluations of health care providers as the main users of mHealth apps are also critical. Thus, we translated the original MAUQ (interactive apps for health care providers) into Chinese and validated the modified questionnaire in our study. This study represents the first cross-cultural adaptation and validation of the MAUQ for interactive apps for health care providers into Chinese, addressing a critical gap in usability assessment tools for medical professionals in China’s mHealth landscape.

The Chinese adaptation process of the health care provider version of the MAUQ was standardized. Before the Chinese adaptation of this scale, authorization was obtained, and the preparatory work was standardized. The translation model by Brislin [17] was strictly followed. The translators and back translators all had a master’s degree or above. For the disputed items in each version, decisions on modification were made through collective discussions by the research group, so the translation process was standardized and accurate. After the initial Chinese-adapted scale was developed, experts in the fields of medicine and English were invited to conduct cross-cultural adjustments from different professional perspectives. Revisions were made while ensuring the equivalence of the Chinese-adapted scale and the original scale to the greatest extent so as to ensure accuracy, comprehensibility of the item content, and its consistency with the local culture and further improve the adaptability of the scale. Then, a preliminary validation was carried out among medical staff. Methods such as item analysis and reliability and validity tests were used to test the internal consistency and effectiveness. The results of the presurvey study showed that the items of this Chinese-adapted scale were clear, which was convenient for the research participants to understand and answer. Therefore, the Chinese adaptation process of this scale was scientific and standardized, and the results were reliable.

Reliability evaluation can reflect the stability and consistency of the scale. In this study, the Cronbach α was used for consistency testing, and test-retest reliability was used for stability testing. The results showed that the Cronbach α of the MAUQ-C for health care providers was 0.919 (>0.8). The Cronbach α values of the 3 dimensions of the scale were 0.928, 0.877, and 0.886, all above 0.8, indicating that the Chinese-adapted scale had good internal consistency. The overall test-retest reliability of the scale was 0.881, and the test-retest reliability of each dimension was 0.759, 0.874, and 0.745, all above 0.7, indicating that this Chinese-adapted scale had good stability. Validity evaluation can show whether the scale truly and accurately reflects the content it surveys. In this study, methods such as content validity and factor analysis were used to test the validity of the Chinese-adapted scale. The content validity results showed that the I-CVI (0.833-1) of each item and the Scale-Content Validity Index/Average (0.992) of the scale met the standards, indicating that the experts had a high degree of recognition of the Chinese-adapted items and their corresponding measurement content and each item could effectively reflect the usability of the procedure, which indicated that the content validity of the MAUQ-C for health care providers was good. EFA extracted 3 common factors, which were consistent with the dimension division of the original scale, indicating that the item results were reasonable and the structural validity was good. The CFA results showed that the model fit met the standards (minimum discrepancy/df=1.810; root-mean-square error of approximation=0.058; incremental fit index=0.950; comparative fit index=0.950; goodness-of-fit index=0.887; normed fit index=0.895; relative fit index=0.881), indicating that the Chinese-adapted scale was consistent with the original scale in structure. These psychometric properties are consistent with previous validation studies of the MAUQ in other languages [8,11,15]. For instance, the 3-factor structure identified in our study aligns with the original development study by Zhou et al [12], confirming that the usability dimensions—usability and satisfaction, system information arrangement, and efficiency—are robust across different cultural and linguistic contexts. This cross-cultural stability suggests that the MAUQ-C is a reliable instrument for measuring usability among Chinese health care providers, offering a solid foundation for further research in this population.

Owing to the rapid development of the internet, mHealth apps have also developed rapidly. As the usability evaluation of apps can effectively identify and correct problems in and thereby optimize their operation, this method has become a common step in the app development process. In addition, the usability of apps also affects patients’ use experience and the final intervention effect. Therefore, scholars at home and abroad have paid more and more attention to the usability evaluation of mHealth apps in recent years [10]. Unlike widely used instruments such as the SUS, Software Usability Measurement Inventory, or Questionnaire for User Interaction Satisfaction—which focus on technical usability for general software or websites—the MAUQ is uniquely tailored to mHealth contexts. Its differentiation between patient and health care provider versions and emphasis on interactive app functionalities addresses the specialized needs of health care professionals, whose perspectives are critical for optimizing the clinical workflow integration of mHealth tools.

The robust psychometric properties of the MAUQ-C suggest that usability is not a monolithic construct but is highly context dependent. Unlike general usability scales (eg, the SUS), the MAUQ-C’s focus on system information arrangement and efficiency reflects the specific cognitive and operational demands placed on health care providers. The mechanism underlying this validity lies in the instrument’s alignment with the clinical workflow; when an app reduces the cognitive load of data entry or information retrieval, app users perceive higher usability. This study’s original contribution is that, first, it provides a psychometrically sound instrument tailored to the Chinese clinical environment and, second, it shifts the focus of mHealth evaluation from generic technical performance to professional-specific utility, which is a prerequisite for the successful integration of digital health into routine clinical practice.

Debates in mHealth usability research have highlighted the tension between subjective self-reported measures and objective behavioral metrics. While objective data (eg, task completion time and error rates) provide precise insights into technical performance, they often fail to capture the user’s cognitive load, satisfaction, and perceived workflow integration—dimensions that are critical for long-term app adoption in clinical settings. Our study contributes to this debate by demonstrating that the MAUQ-C provides a reliable subjective assessment that complements rather than replaces objective performance data. However, we acknowledge that relying solely on self-reported instruments may be subject to recall and social desirability bias. Future research should aim to triangulate MAUQ-C scores with objective use logs to provide a more holistic view of mHealth usability.

Unlike previous system usability evaluation tools, the MAUQ was not only developed for mHealth apps. Its 4 original versions are also carefully divided according to whether the app interacts with patients, and the evaluation objects are divided into 2 categories: patients and medical staff, which is more targeted. While the patient version of the MAUQ has been adapted into Chinese, this study fills the unmet need for a validated health care worker–specific tool. This is significant because health care providers serve as both end users and intermediaries in mHealth interventions, making their usability feedback essential for enhancing app adoption and effectiveness in clinical settings.

### Limitations

This study has several limitations. First, the evaluation was restricted to an app for populations with chronic diseases; future research should validate the MAUQ-C across diverse mHealth contexts to ensure broader generalizability. Second, the use of convenience sampling in 2 hospitals may introduce selection bias; thus, multicenter randomized studies are recommended to enhance representativeness. Third, the cross-sectional design precludes the assessment of long-term usability or the impact of app updates; longitudinal studies are needed to capture evolving user perceptions. In addition, conducting EFA and CFA on the same dataset may have inflated model fit indexes. Future validation should use a split-sample approach or independent datasets to provide a more rigorous confirmation of the factor structure. Finally, this study lacks an assessment of convergent and discriminant validity. Future research should compare the MAUQ-C with established usability instruments such as the SUS or the PSSUQ to further demonstrate its unique utility and construct validity in measuring mHealth usability for health care professionals.

### Conclusions

The MAUQ-C for interactive apps for health care providers is a psychometrically sound and culturally adapted tool ideal for assessing mHealth app usability in Chinese clinical settings. Its validation advances mHealth research and practice by providing health care professionals, developers, and researchers with a reliable instrument to drive user-centered design and improve the quality of digital health interventions.

## Abbreviations

CFA: confirmatory factor analysis
CVI: content validity index
EFA: exploratory factor analysis
KMO: Kaiser-Meyer-Olkin
MAUQ: mHealth App Usability Questionnaire
MAUQ-C: Chinese version of the mHealth App Usability Questionnaire
mHealth: mobile health
PSSUQ: Post-Study System Usability Questionnaire
SUS: System Usability Scale

## Data Availability

The datasets generated and analyzed during the current study are available from the corresponding author on reasonable request.
